# Plasma Amino Acid Profile in Patients with Aortic Dissection

**DOI:** 10.1038/srep40146

**Published:** 2017-01-10

**Authors:** Linlin Wang, Sha Liu, Wengang Yang, Haitao Yu, Li Zhang, Ping Ma, Peng Wu, Xue Li, Kenka Cho, Song Xue, Baohong Jiang

**Affiliations:** 1 Shanghai Institute of Materia Medica, Chinese Academy of Sciences, Shanghai 201203, China; 2 Department of Cardiovascular Surgery, Renji Hospital, Shanghai Jiaotong University School of Medicine, Shanghai 200127, China; 3 Covidien (Shanghai) Management Consulting Co., Ltd, Shanghai 200233, China; 4 The Second Artillery General Hospital PLA, Beijing 100088, China; 5 Takarazuka University of Medical and Health Care, Hanayashiki-Midorigaoka, Takarazuka-city 6660162, Japan

## Abstract

Aortic dissection (AD), a severe cardiovascular disease with the characteristics of high mortality, is lack of specific clinical biomarkers. In order to facilitate the diagnosis of AD, we investigated plasma amino acid profile through metabolomics approach. Total 33 human subjects were enrolled in the study: 11 coronary heart disease (CHD) patients without aortic lesion and 11 acute AD and 11 chronic AD. Amino acids were identified in plasma using liquid chromatography and mass spectrometry (LC-MS/MS), and were further subjected to multiple logistic regression analysis. The score plots of principal component analysis (PCA) and partial least squares-discriminate analysis (PLS-DA) showed clear discrimination of CHD patients with AD, acute AD or chronic AD patients, respectively. The contents of histidine, glycine, serine, citrate, ornithine, hydroxyproline, proline and sarcosine were significant different in acute AD patients comparing with CHD patients. The levels of citrate, GABA, glutamate and cysteine were significant different in chronic AD patients comparing with CHD patients. The contents of glutamate and phenylalanine were significant changed in acute AD patients comparing with chronic AD patients. Plasma aminograms were significantly altered in patients with AD comparing with CHD, especially in acute AD, suggesting amino acid profile is expected to exploit a novel, non-invasive, objective diagnosis for AD.

Aortic dissection (AD) is a potentially lethal vascular disease with the characteristics of high mortality^1^. AD begins as a spontaneous tear through either the intima or the adventitia that extends into the media of the aortic wall, and the torn aorta is prone to dilatation and fatal aortic rupture. If left untreated, the mortality rate of AD can reach 50–60% within 48 hours and 80% within two weeks^2^, so timely diagnosis is fundamental to save lives.

At present stage, the diagnosis of AD was mainly made according to confirmatory imaging such as spiral computed tomography angiogram^3^, magnetic resonance imaging^4^ or intraoperative visualization such as transthoracic or transesophageal echocardiography^5^. These imaging detection are costly and frequently require patient transfer to specialized clinical centers. Furthermore, computed tomography angiogram, the most frequently used imaging detection for suspected AD, exposes patients to significant radiations and carries inherent risks of anaphylaxis and medium contrast nephropathy. Hence, development of circulating markers that can signal the onset of AD timely would be needed to assist physicians to diagnosis and further to rescue the lives of AD patients.

Among metabolites, the amino acid balance in patients with various diseases often differs from that maintained in healthy individual. Overall, 20% of the human body is composed of amino acids and their metabolites, which play important roles as both basic substrates and regulators in many metabolic pathways^6^. Specific abnormalities in plasma amino acids concentrations have been reported in human liver fibrosis^7^ and non-small cell lung cancer^8^. Plasma amino acid profiles were also used to discriminate patients with breast cancer, esophageal cancer, head and neck cancers, from healthy controls^9^,^10^,^11^. A significant increase in free tryptophan has been reported in lung cancer patients^12^. Therefore, metabolic changes detecting from amino acid profiles could potentially be useful in disease diagnosis.

Profiling analysis is a form of quantitative analysis aimed at a few preselected metabolites, and is one of a number of metabonomic research strategies^13^. Post-genomic technologies offer possibilities for exploiting amino acids profiling, especially the methods for amino acids analysis have been established using a rapid and highly performance liquid chromatography-tandem mass spectrometry (HPLC-MS/MS)^14^,^15^. New technology development reduced both the time and the cost of analysis for amino-acid measurements.

Severe pain was the most common presenting symptom and the majority of AD patients complained of chest pain which is similar with the onset of coronary artery disease^16^. Therefore; we investigated the possibility to use amino acid profile for discrimination of aortic dissection from coronary artery disease, and to further develop amino acid profile as a novel diagnostic method to save the lives of AD patents in time.

## Methods

### Patients

Enrolment was the patients from December 2010 to March 2012, who attended the Department of Cardiovascular Surgery of Renji Hospital, Shanghai Jiaotong University School of Medicine, China. In this setting, 22 consecutive AD patients were selected (AD group) on the basis of the following inclusion criteria: 1) type A Stanford dissection, 2) type B Stanford dissection, 3) no history of neoplasm, autoimmune or inflammatory systemic disease, 4) no presence of known genetic syndromes that cause aortic disease, including Marfan syndrome, or family history of aortic dissection or aneurysm. 11 CHD subjects were selected (CHD group), as follows: 1) coronary heart disease, 2) no history of vascular disease, 3) no presence of known genetic syndromes that cause CHD or family history of CHD, 4) no history of neoplasm, autoimmune or inflammatory systemic disease.

All written informed consent was obtained from each patient or family member before inclusion in this study. The Medicine Ethics Committee of Renji Hospital approved the study protocol, which also conformed to the principles of the Helsinki Declaration.

### Assessment of Disease Type

Diagnosis of AD was confirmed using standard criteria by computed tomography (CT). The data were acquired using an ECG-gated dynamic 64-slice CT scanner (Sensation 64, Siemens Medical Solutions, Forchheim, Germany). Images were acquired during a single breath-hold phase of 20 seconds, during which the entire chest and abdomen were imaged. Analysis of the dynamic scans was performed using General Electric Discovery HD 750 scanner (GE Healthcare, USA). All the patients in whom the onset of AD could be clearly determined (e.g., by symptoms) were included. The AD patients were separated into acute and chronic-stage. Acute AD indicated that blood samples were drawn within the first 48 hours after dissection onset; chronic AD indicates that blood samples were drawn after 14 days from dissection onset.

### Collection of Blood Samples

Peripheral venous blood samples were collected and immediately underwent plasma isolation. The blood was immediately collected into vacuette™ tubes (Greiner Bio-One, Frickenhausen, Germany). Sample was centrifuged 1000 g for 10 min at room temperature (L-500 centrifuge, Xiang-Yi, China). The plasma was then separated into aliquots in cryogenic vials (Greiner Bio-One, Frickenhausen, Germany) and stored in liquid nitrogen before analysis of amino acids as described below. Quality control (QC) samples were prepared by pooling and mixing 10 μL the sample volume of each sample.

### Clinical Chemistry Measurements

Plasma biochemical analyses were carried out on an AMS-18 automatic biochemistry analyzer (Beijing Option Science&Technology Development Co., Ltd., Beijing, China) including Urea, creatinine (Cr), sodium (Na) and potassium (K). An independent sample *t*-test was conducted to compare the clinical biochemical data of CHD with acute AD, chronic AD or total AD, respectively. Furthermore, the independent sample t-test was also performed to compare the difference between acute AD and chronic AD.

### Sample Preparation for LC-MS/MS Analysis

Samples were measured with a TRAQ^TM^ kit (AB Sciex Pte. Ltd). An aliquot (40 μL) of plasma sample was a mixed with sulfosalicylic acid (10 μL) in a tube, vortexed for 30 s at room temperature, then centrifuged (10000 g, 2 min, 4 °C) to deposit protein following. The supernatant (10 μL) was transferred into a clean tube and mixed with 40 μL borate buffer (5 mmol/L, PH 8.5). Each sample was then vortexed vigorously for 30 s at room temperature followed by centrifugation at 10000 g for 2 min. 10 μL supernatant was transferred into another tube, mixed with 5 μL iTRAQ reagent (AB Sciex Pte. Ltd), then vortexed and centrifuged. The tubes were incubated for at least 30 min at room temperature. And then the 5 μL NH_2_OH was added to each tube for mix and centrifugation. At last, 32 μL internal standard was added to each tube and then vortexed and centrifuged. The processing method of QC was consistent with that of plasma samples.

### LC-MS/MS Parameters

Prepared samples were analyzed on a Dionex Ultimate 3000 HPLC (Dionex AG, Switzerland) which paired with a 3200 Q-TRAP mass spectrometer (AB Sciex, USA). In brief, 3 μL of each sample was injected on an AAA C18 column (150 × 4.6 mm, 5 μm particle size) at 50 °C. The mobile phase consisted of water including 0.1% formic acid (solvent A) and acetonitrile including 0.1% formic acid (solvent B). The gradient for the amino acid elution switched from 2% solvent B to 28% solvent B after 10 min; ascended to 100% solvent B in 0.1 min and maintained for 5.9 min; backed to 2% solvent B in 0.1 min and maintained for 3.9 min. The flow rate was 0.8 mL/min.

Nitrogen was used as curtain and nebuliser gas at the pressure of 20 psi (Curtain), 55 psi (GS1), and 60 psi (GS2), respectively. Desolvation temperature (TEM) was maintained at 580 °C, with the respective voltages: 5500 V (IS), 35 V (DP), 10 V (EP), 5 V (CXP). QC samples were analyzed at every 6 injections to monitor the instrument stability. We transformed the spectral map into the content of each amino acid, and saved in Excel file format. And then it was used for pattern recognition.

### Pattern Recognition and Statistical Analysis

Multivariate data analysis was conducted with the software SIMCA-P^+^ 12.0 (Umetrics, Sweden). Principal component analysis (PCA) was performed to visualize general clustering, trends and outliers. Partial least squares projection on latent structure-discriminant analysis (PLS-DA) was subsequently conducted using the par-scaling LC-MS/MS data.

Potential markers were extracted from loading scatter plots, and the heat map was analyzed in-house by Matlab script (V7.0, Math-works, MA). In order to further select differential variables in the PLS-DA models, the cutoff value of 1 was set in the variable importance in the projection (VIP). The selected variables between AD patients and CHD controls were then performed using one way analysis of variance (ANOVA) with Tukey HSD analysis as implemented in IBM SPSS Statistics (19.0) software, and *p* < 0.05 was considered significant.

## Results

### Baseline Characteristics of Patients

The baseline characteristics of patients were mainly evaluated by plasma biochemical analysis and presented in Table 1. All the values of body mass index (BMI) were significant higher for acute AD patients (29.60 ± 7.59 kg/m^2^, p < 0.05), chronic AD patients (26.95 ± 4.29 kg/m^2^, p < 0.05) or AD patients (28.1 ± 5.9 kg/m^2^, p < 0.05) comparing with CHD patients (22.0 ± 3.3 kg/m^2^), respectively. The decline of potassium (K) level was found for acute AD (3.69 ± 0.28 mEg/L, p < 0.01), chronic AD (3.95 ± 0.37 mEg/L, p < 0.05) or AD (3.82 ± 0.34 mEg/L, p < 0.01) comparing with CHD patients (4.33 ± 0.39 mEg/L), separately. The decline of K level was also found for acute AD comparing with chronic AD ((3.69 ± 0.28 mEg/L versus 3.95 ± 0.37 mEg/L, p < 0.05). No differences were detected on age, sex, systolic blood pressure (SBP), urea, creatine (Cr) or sodium (Na) between any two groups among acute AD, chronic AD, total AD and CHD patients.

### LC-MS/MS Spectra of Amino Acids from Plasma

**Figure 1:**
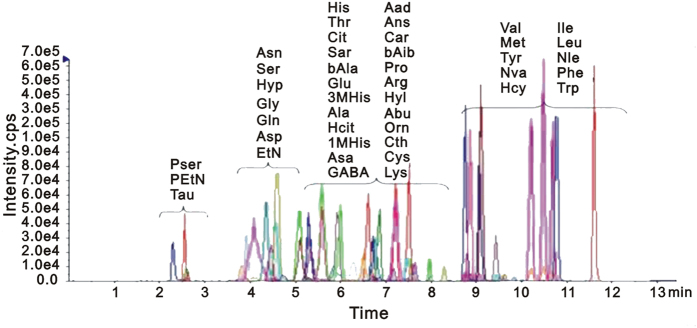
Overlaid chromatogram of the marker amino acids detected by multiple reaction monitoring (MRM).

A robust and sensitive quantitative method for profiling amino acids was used. The total ion chromatograms of all samples demonstrated a strong signal, and reproducible retention time, indicating the reliability of metabolomic analysis. These amino acids were verified by ion fragment patterns and retention time through comparing with standard amino acids. Overlaid chromatogram of the marker amino acids detected by multiple reaction monitoring (MRM) was shown in Fig. 1. Obvious chromatographic differences were observed between different group samples, and a total of more than 40 amino acids were identified. These amino acids were dominated by phospho-serine (Pser), phospho-ethanolamine (PEtN), taurine (Tau), asparagines (Asn), serine (Ser), hydroxyproline (Hyp), glycine (Gly), glutamine (Gln), asparate (Asp), ethanolamine (EtN), histidine (His), threonine (Thr), citrate (Cit), sarcosine (Sar), β-alanine (bAla), glutamate (Glu), 3-Methyl-histidine (3M-His), alanine (Ala), homocitrulline (Hcit), 1-Methyl-histidine (1M-His), argininosuccinic acid (Asa), γ-amino-n-butyrate (GABA), α-amino adipic acid (Aad), anserine (Ans), carnosine (Car), β-amino isobutyric (bAib), proline (Pro), arginine (Arg), hydroxylysine (Hyl), α-amino-n-butyrate (Abu), ornithine (Orn), cysteine (Cys), lysine (Lys), valine (Val), methionine (Met), tyrosine (Tyr), norvaline (Nva), homocystine (Hcy), isoleucine (Ile), leucine (Leu), norleucine (Nle), phenylalanine (Phe) and tryptophan (Trp).

The data for system repeatability and stability were listed in Table S1 (ESI+). The RSD of amino acids in QC samples were all between 0.00–14.61%, which indicated that the established sample analysis method was repeatable and stable, and could be used for metabolomics experiments.

### AD and CHD Patients Were Distinguished by PCA Analysis

**Figure 2:**
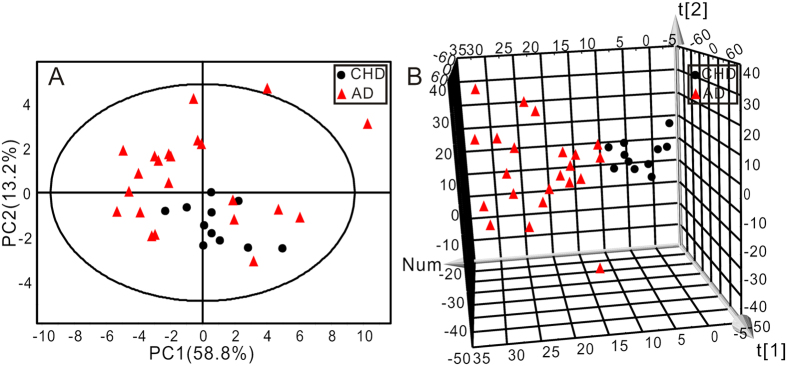
The score plots of two-dimensional (**A**) and three-dimensional (**B**) PCA showed the changes in the human plasma amino acids data from CHD (●) and AD (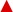) groups (R^2^X = 68.7%, Q^2^ = 45.4%).

PCA was first performed to get an overview of the difference on plasma amino acid profile between CHD and AD patients. The AD group contained both acute AD and chronic AD patients. The two-dimensional (2D) score plot of PCA was shown in Fig. 2A and the three-dimensional (3D) score plot of PCA was shown in Fig. 2B. The 2D score plot of PCA analysis clustered but not separated the two groups, while 3D score plot distinguished the two groups more significantly.

**Figure 3:**
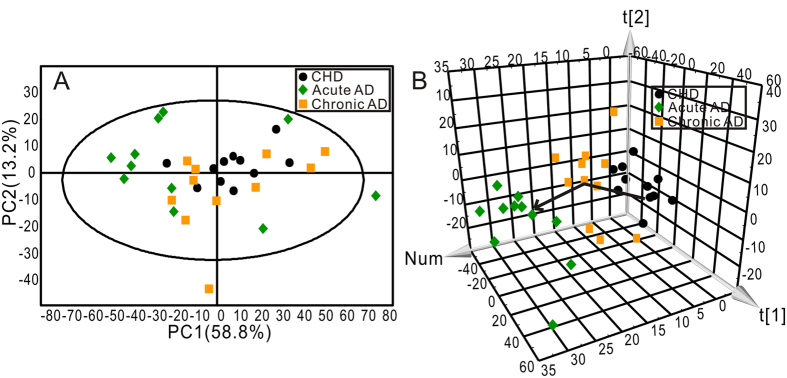
The score plots of two-dimensional (**A**) and three-dimensional (**B**) PCA showed the changes in the human plasma amino acids data from three groups. CHD group (●), Acute AD group (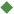), Chronic AD group (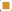) (R^2^X = 72.0%, Q^2^ = 48.3%).

To further evaluated the change of amino acids profile with AD progression, AD group was subdivided into acute AD and chronic AD, and then PCA analysis was carried out following. The 2D score plot of PCA was shown in Fig. 3A and the 3D score plot of PCA was shown in Fig. 3B. The three subject groups could not be distinguished by 2D score plot of PCA analysis but an obvious divergent trajectory was revealed based on 3D score plot among three groups. The cluster of acute AD located farther away from CHD, and the cluster of chronic AD located between acute AD and CHD.

### AD and CHD Patients Were Distinguished by PLS-DA Analysis

**Figure 4:**
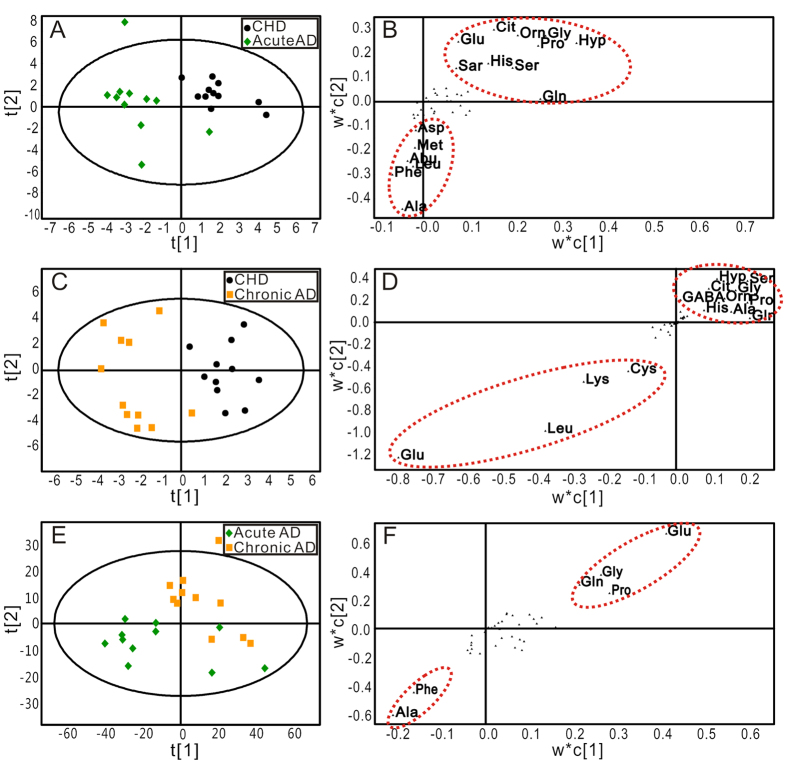
The score and loading plots of PLS-DA for the plasma samples in different groups. (**A**,**B**) PLS-DA score and loading plots of samples between CHD and Acute AD groups (R^2^X = 68.7%, Q^2^Y = 70.7%, Q^2^ = 42.9%); (**C**,**D**) PLS-DA score and loading plots of samples between CHD and Chronic AD groups (R^2^X = 46.0%, Q^2^Y = 89.2%, Q^2^ = 13.2%); (**E**,**F**) PLS-DA score and loading plots of samples between Acute AD and Chronic AD groups (R^2^X = 73.7%, Q^2^Y = 65.1%, Q^2^ = 39.6%). CHD group (●), Acute AD group (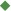), Chronic AD group 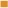).

To achieve the maximum distinction and identify differential amino acids that accounted for the separation between groups, PLS-DA was conducted following PCA. The score plots of PLS-DA for acute AD and CHD (Fig. 4A), chronic AD and CHD (Fig. 4 C), and for acute AD and chronic AD (Fig. 4E) were shown, respectively. Acute AD or chronic AD was clearly separated from the CHD group, which holds a higher efficiency to distinguish between groups comparing with PCA score plots (Fig. S1 A and B). However, acute AD was not properly separated from chronic AD based on score plot of PLS-DA. The robustness of these PLS-DA classification models was assessed by 200 times permutation tests. The *R*^2^ and *Q*^2^ values were lower than the original ones, and the blue regression lines of the *Q*^2^-points intersected the vertical axis below zero, implying the validation of these PLS-DA models (Fig. S2)^17^.

The scatter loading plots of the PLS-DA, which was a pairwise comparison model, displayed the altered amino acids between two groups. Comparing with CHD patients, the levels of alanine, phenylalanine, leucine, α-amino-n-butyrate, methionine and asparate were up-regulated, while the levels of hydroxyproline, glutamine, glycine, proline, ornithine, citrate, glutamate, sarcosine, histidine and serine were down-regulated in acute AD patients (Fig. 4B). Comparing with CHD patients, the levels of glutamate, leucine, lysine and cystine were up-regulated in chronic AD patients, and the levels of hydroxyproline, serine, citrate, glycine, GABA, ornithine, proline, histidine, alanine and glutamine were down-regulated in chronic AD patients (Fig. 4D). Comparing with chronic AD patients, the level of phenylalanine was up-regulated and the level of glutamate was down-regulated in chronic AD patients (Fig. 4F).

### Amino Acids Metabolites in AD Patients Were Identified

**Figure 5:**
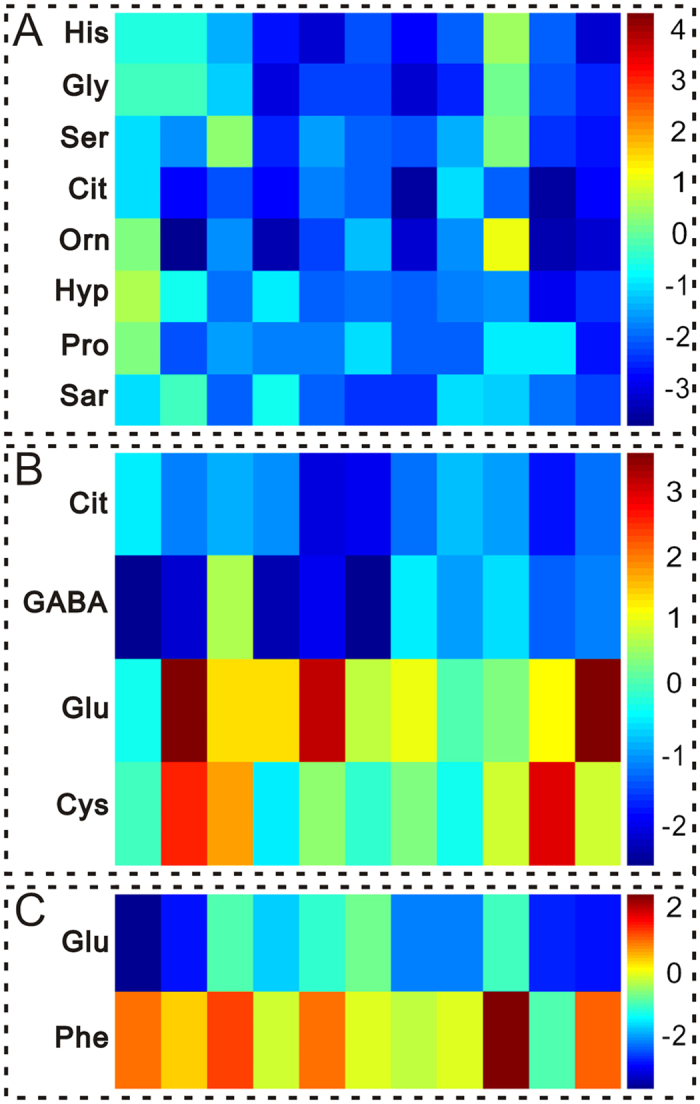
Heat-map for key amino acids change color-scaled with z-scores for the human plasma where the warm (red) colored bars denote elevation of amino acids levels, and cold colored (blue) ones indicate a decrease compared to the CHD patients. Every amino acid is delegated by a single row of colored boxes, but columns correspond to different samples. Each value was standardized on the basis of the controls (**A**,**B**) CHD patients; (**C**) Chronic AD)) with subtracting mean and dividing by the standard deviation of controls (CHD patients). (**A**) Acute AD group versus CHD patients; (**B**) Chronic AD group versus CHD patients. (**C**) Acute AD group versus Chronic AD group.

The changed amino acids in scatter loading were only been preliminary screened. To further screen out the potential amino acids that specifically indicated AD disease and further AD progression, we calculated the variable importance for the projection (VIP) values from the PLS-DA models. PLS-DA models signified the influence of the amino acids on the classification, and the VIP values > 1.0 represented that the changed amino acids contributed significantly to the clustering. Only those amino acids (VIP > 1.0) with significant difference (*P* < 0.05) between AD and CHD, or between acute AD and chronic AD groups were selected as potential candidate using one way analysis of variance. Under the criteria of VIP > 1.0 and *P* < 0.05, 8 amino acids in acute AD and 4 amino acids in chronic AD were identified as potential candidates differentiating AD from CHD. With the same criteria, 2 amino acids were further identified as potential candidates differentiating acute AD from chronic AD (Table 2). To visualize the alterations of the potential amino acids, a heat-map was further generated based on Z-score values (Fig. 5). Among these amino acids identified in acute AD and chronic AD patients, citrate was the only amino acid that altered in both two groups. Comparing with CHD patients, the down-regulation of histidine, glycine, serine, ornithine, hydroxyproline, proline and sarcosine in acute AD group, and GABA in chronic AD patients were detected, and the up-regulation of glutamate and cystine were recognized in chronic AD patients. The decreased level of glutamate and the increased level of phenylalanine were recognized in acute AD patients comparing with chronic AD patients.

## Discussion

AD, especially acute AD, is a life-threatening disease that is easily misdiagnosed In the present study, citrate, histidine, glycine, hydroxyproline, proline, serine and cysteine were specific regulated in AD patients comparing with CHD patients. Glutamate and phenylalanine showed differential pattern in acute AD comparing with chronic AD, suggesting both diagnosis and prognostic value of aminogram for AD patients.

**Figure 6:**
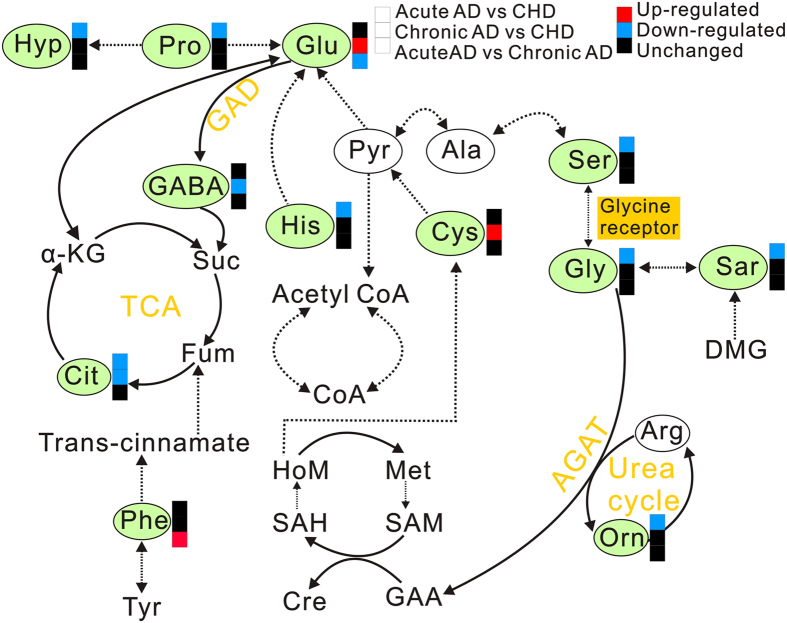
The metabolic pathways of the most disregulated amino acids in plasma of AD patient. The tag in green represents the significant change of amino acids. The red boxes denote significant up-regulation of amino acids in Acute AD and Chronic AD groups compared with CHD group, while the blue boxes represent significant down-regulation. The black boxes denote no significant change.

Tricarboxylic acid cycle (TCA cycle), also known as citric acid cycle or Krebs’ cycle, consists of a series of chemical reactions aimed at generating energy (ATP) through oxidation of acetate from carbohydrates, fats and proteins into carbon dioxide and water. The plasma levels of citrate, the important intermediate in TCA cycle, were down-regulated in both AD patients (acute AD and chronic AD) (Fig. 6), indicating the dys-regulation of TCA cycle and impairment on mitochondrial function in both acute AD and chronic AD patients^18^,^19^,^20^.

As a semi-essential amino acid, histidine is necessary for infant and uremic patients^21^. Histidine can be converted to glutamate, and then via deamination to produce α-ketoglutaric acid for use in TCA cycle (Fig. 6). However, the level of glutamate was no significant change in acute AD group, so the down-regulation of histidine was not a prerequisite for the inhibition of TCA. On the other hand, histidine can be converted into histamine under the action of histidine decarboxylase, and histamine shows strong effect on vasodilation^22^. In our study, the down-regulated histidine in acute AD patients may reduce the formation of histamine and further influence the function of aorta. The detail regulation of histidine-histamine pathway and its role in AD need more research to evaluate.

Glycine is synthesized from threonine through inter-organ metabolism of amino acids (Fig. 6), and it is also the precursor of many important metabolites (e.g., creatine, purines, heme and glutathione). Serine hydroxymethyl transferase converts serine into glycine and *vice versa* (Fig. 6). And sarcosine can be converted into glycine by sarcosine dehydrogenase (Fig. 6)^23^. The decrease on the content of serine and sarcosine in acute AD patients may possibly become one of the important factors inducing the decline of glycine. Glycine exhibits sorts of biological characteristics, containing anti-oxidative, metabolic regulation, immunomodulatory and cytoprotective characteristics^24^,^25^,^26^,^27^. In response to diseases and malnutrition such as diabetes and starvation, plasma glycine significantly decreased^28^,^29^. Gómez-Zamudio and co-workers also demonstrated that glycine could improve the endothelium function in aged rats^30^. In our study, glycine declined in acute AD patients, potentially indicating the possible relation between endothelium dysfunction and glycine metabolism disorder.

Hydroxyproline is generated by proline through proline hydroxylase, and proline is a peculiar amino acid in collagen (Fig. 6)^31^. Collagen synthesis requires posttranslational modifications of procollagens, including hydroxylation^32^ Collagen is the key support material to maintain vascular structures. As an important component of collagen, the level of proline was down-regulated in acute AD patients. Proline deficient might induce obstacles to collagen synthesis, and further led to the reconstruction and damage on the vascular structures.

Remarkably, the change of cysteine is specific in chronic AD patients. Cysteine can be directly taken off sulfydryl and amino to generate pyruvate, which can form acetyl-CoA through oxidative decarboxylation, and then be further converted to glutamate (Fig. 6). As a metabolite in homocysteine metabolism, the cysteine and homocysteine play vital roles in living organism^33^. Homocysteine is the amino acid directly related to vessel disease. Furthermore, homocysteine was also increased not only in Marfan patients, but also increased in patients with acute aortic dissection and chronic thoracic aoryic aneurysms^34^,^35^,^36^ Plasma homocysteine exists in three major forms, with trace amounts (1%) existing in the reduced form. Approximately 70% is bound to albumin, and the remaining 30% forms low molecular weight disulfides predominantly with cysteine. The sum of all these homocysteine species is termed total homocysteine (tHcy). Reducing reagent is necessary for plasma treatment to detect the concentration of homocysteine, while we were concerned to detect more amino acids with significant changes for AD patients and did not perform special treatment on plasma samples. We hope to optimize the experimental conditions to catch more amino acids with significant change in the future.

Through metabolomic analysis, we found that the significant changed amino acids were histidine, glycine, serine, citrate, ornithine, hydroxyproline, proline and sarcosine in acute AD patients; and citrate, GABA, glutamate and cysteine in chronic AD patients. The significant changed amino acids were glutamate and phenylalanine in acute AD patients comparing with chronic AD patients. These amino acids play an important role in maintaining the stability of energy metabolism, endothelial function and vascular structure, indicating that the amino acid profile is expected to provide a powerful support for the diagnosis and prognosis of AD.

## Conclusions

In conclusion, our work showed the significant distinct amino acids profiles in plasma samples from AD, acute AD or chronic AD comparing with CHD, as well as between acute AD and chronic AD; suggesting that amino acids profiling could possibly provide a sensitive, feasible diagnostic value for AD patients.

## Additional Information

**How to cite this article**: Wang, L. *et al*. Plasma Amino Acid Profile in Patients with Aortic Dissection. *Sci. Rep.* **7**, 40146; doi: 10.1038/srep40146 (2017).

**Publisher's note:** Springer Nature remains neutral with regard to jurisdictional claims in published maps and institutional affiliations.

## Supplementary Material

Supplementary Materials

## Figures and Tables

**Table 1:** The clinical data for the human plasma samples.

Clinical indicator	Acute AD	Chronic AD	AD	CHD
Sex (M/F)	10/1	10/1	20/2	10/1
Age (year)	48 ± 14	52 ± 10	50 ± 12	50 ± 15
BMI (kg/m^2^)	29.60 ± 7.59^*^	26.95 ± 4.29^*^	28.1 ± 5.9^**^	22.0 ± 3.3
SBP (mmHg)	129.0 ± 17.71	134.18 ± 26.18	131.59 ± 21.97	118.91 ± 21.27
Urea (mmol/L)	7.16 ± 1.94	7.09 ± 2.25	7.12 ± 2.05	6.73 ± 2.12
Cr (μmol/L)	75.36 ± 17.82	72.05 ± 20.50	73.62 ± 18.82	71.28 ± 13.53
Na (mEq/L)	140.30 ± 5.23	141.30 ± 2.36	140.80 ± 3.98	141.91 ± 2.88
K (mEq/L)	3.69 ± .028^**#^	3.95 ± 0.37^*^	3.82 ± 0.34^**^	4.33 ± 0.39

Values are presented as mean ± SD. ^*^Indicates significant changes compared with CHD ^*^p < 0.05; ^**^p < 0.01. ^#^Indicates significant changes from Acute AD vs. Chronic AD ^#^*p* < 0.05; ^##^*p* < 0.01. BMI: body mass index; SBP: systolic blood pressure; Cr: creatine. Na: sodium; K: potassium.

**Table 2:** Identified differential amino acids in human plasma.

Amino acids	Acute AD versus CHD			Chronic AD versus CHD			Acute AD versus Chronic AD		
	VIP	*P*	FC	VIP	*P*	FC	VIP	*P*	FC
His	1.30	0.022	0.82	1.13	/	0.90	/	/	0.91
Gly	1.31	0.033	0.77	/	/	0.94	/	/	0.82
Ser	1.15	0.053	0.79	1.11	/	0.87	1.00	/	0.91
Cit	1.85	0.001	0.57	1.81	0.005	0.74	/	/	0.77
Orn	1.30	0.020	0.69	/	/	0.88	/	/	0.78
Hyp	1.28	0.022	0.58	1.28	/	0.69	/	/	0.84
Pro	1.29	0.009	0.73	/	/	0.94	1.26	/	0.78
Sar	1.36	0.027	0.60	1.16	/	0.79	/	/	0.76
GABA	1.59	/	0.92	1.59	0.011	0.76	/	/	1.21
Glu	1.42	/	0.89	1.59	0.003	1.77	2.26	0.014	0.50
Cys	/	/	1.08	1.50	0.016	1.67	/	/	0.65
Phe	/	/	1.15	/	/	0.93	1.67	0.031	1.25

Variable important in projection (VIP) was obtained from PLS-DA with a threshold of 1.0; p-value was calculated from one way ANOVA; Fold change (FC) was calculated as the ratio of the mean amino acid levels between two groups.
