# Supplementary material for: Plasma Amino Acid Profile in Patients with Aortic Dissection
Source: Sci Rep. 2017 Jan 10;7:40146. doi: 10.1038/srep40146 (PMC5223271; doi:10.1038/srep40146)
Supplement: Supplementary Materials [file srep40146-s1.pdf]

Plasma Amino Acid Profile in Patients with Aortic Dissection

Linlin Wang[†,#], Sha Liu[‡,^,#,] Wengang Yang[‡], Haitao Yu[†], Li Zhang[†], Ping Ma[§], Peng Wu[†], Xue Li[†], Kenka Cho[ξ], Song Xue[‡,], Baohong Jiang[†]**

[†]Shanghai Institute of Materia Medica, Chinese Academy of Sciences, Shanghai 201203, China

[‡]Department of Cardiovascular Surgery, Renji Hospital, Shanghai Jiaotong University School of Medicine, Shanghai 200127, China

[^]Covidien (Shanghai) Management Consulting Co., Ltd, Shanghai 200233, China

[§]The Second Artillery General Hospital PLA, Beijing 100088, China

ξTakarazuka University of Medical and Health Care, Hanayashiki-Midorigaoka, Takarazuka-city 6660162, Japan

Corresponding author

Prof. Song Xue, Dongfang Rd#1630, Shanghai, 200127, Chian. Fax: 86 21 58752345.

*E-mail address: xuesong64@163.com

Prof. Baohong Jiang, Haike Rd#501, Shanghai 201203, China. Fax: 86 21 50272223.

*E-mail address: jiangbh@simm.ac.cn.

[#]Authors contributed equally

Fig.S1 PCA scores plots of samples in different groups. A: CHD group (●) vs. Acute AD group (◆) (R^2X=81.6%, Q^2=47.9%); B: CHD group (●) vs. Chronic AD group (■) (R^2X=74.4%, Q^2=33.9%); C: Acute AD group (◆) vs. Chronic AD group (■) (R^2X=78.4%, Q^2=55.9%).

Fig.S1

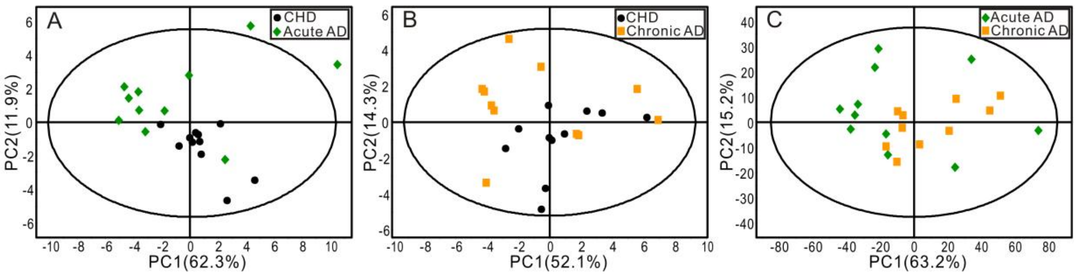

Fig.S2 Permutation test plots (200 permutations) for all the PLS-DA score plots. A: Acute AD vs. CHD. (R^2=0.0, 0.747, Q^2=0.0, -0.225); B: Chronic AD vs. CHD (R^2=0.0, 0.664, Q^2=0.0, -0.089); C: Acute AD vs. Chronic AD (R^2=0.0, 0.409, Q^2=0.0, -0.252).

Fig.S2

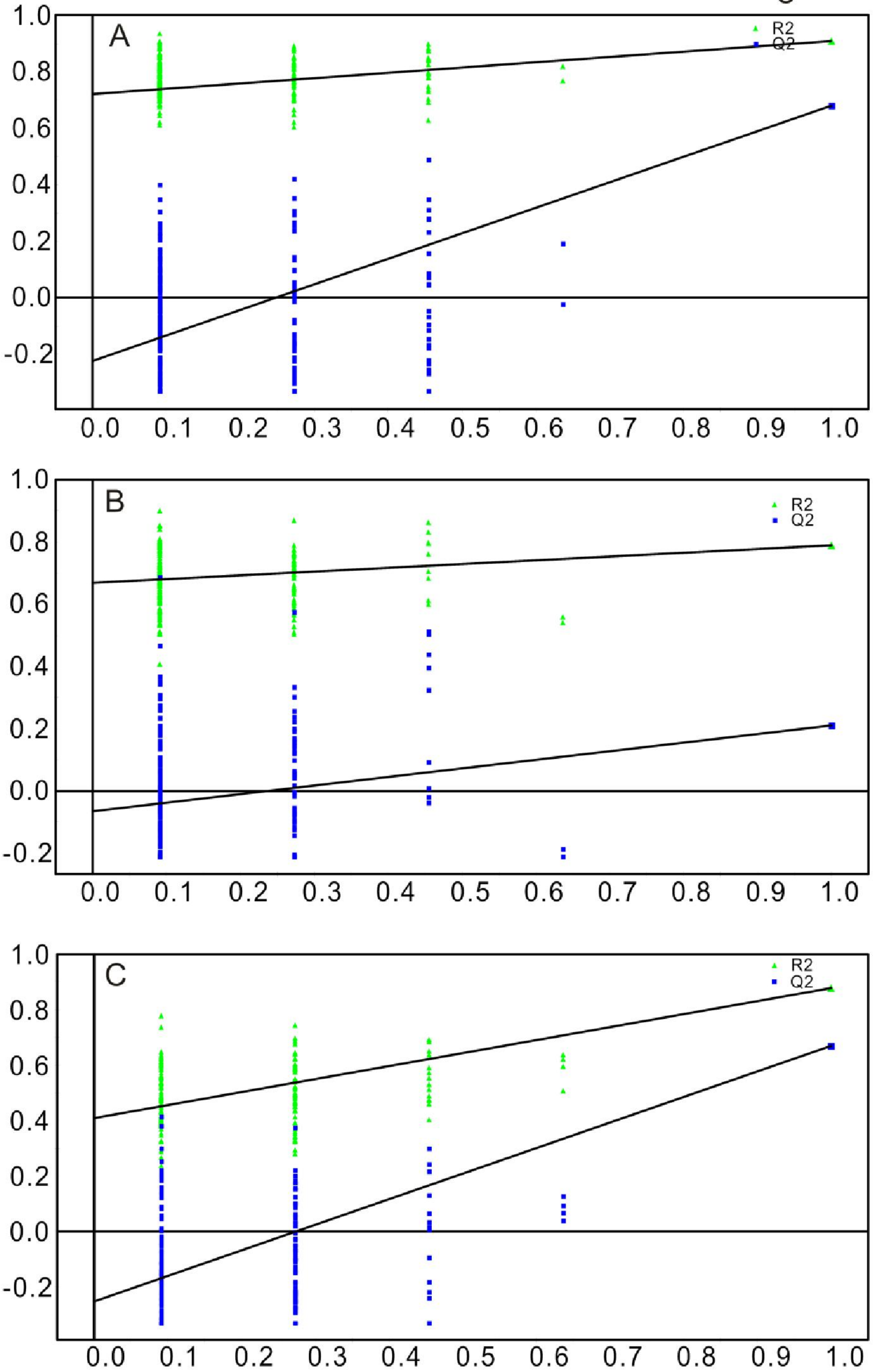

Table S1 Data for quality control samples.

Amino acids	Average	RSD (%)
Arg	47.93	4.91
His	65.73	6.52
Ile	52.70	1.73
Leu	120.92	2.46
Lys	137.10	10.37
Met	16.58	5.36
Phe	49.44	2.84
Thr	110.92	3.23
Trp	35.96	1.82
Val	233.33	1.83
GABA	3.21	10.99
Gly	305.17	5.32
Ser	133.04	4.89
Tau	71.99	9.78
Tyr	43.16	2.97
Aad	0.00	0.00
Asn	42.77	3.71
Asp	3.32	5.34
Cit	36.46	5.83
Glu	97.33	4.29
Gln	504.99	2.15
Orn	83.30	1.94
Cys	16.03	7.57
Cth	0.00	0.00
Hcy	0.00	0.00
Abu	20.65	7.66
Ala	347.66	4.50
Ans	0.00	0.00
bAla	3.83	13.57
bAib	1.18	14.61
Car	0.00	0.00
EtN	8.01	1.48
Hyl	0.00	0.00
Hyp	7.39	7.87
1MHis	6.10	0.31

3MHis	2.45	6.08
PEtN	0.00	0.00
PSer	0.00	0.00
Pro	186.11	8.79
Sar	1.74	9.44
Asa	0.00	0.00
Hcit	0.00	0.00
TOTAL	2796.48	0.43
